# Discovering the Potential of Dental Pulp Stem Cells for Corneal Endothelial Cell Production: A Proof of Concept

**DOI:** 10.3389/fbioe.2021.617724

**Published:** 2021-01-28

**Authors:** Begoña M. Bosch, Enrique Salero, Raquel Núñez-Toldrà, Alfonso L. Sabater, F. J. Gil, Roman A. Perez

**Affiliations:** ^1^Bioengineering Institute of Technology, Universitat Internacional de Catalunya, Barcelona, Spain; ^2^Department of Ophthalmology, Bascom Palmer Eye Institute, University of Miami Miller School of Medicine, Miami, FL, United States; ^3^Imperial College London, National Heart and Lung Institute, London, United Kingdom

**Keywords:** cornea, dental pulp stem cells, neural crest, corneal endothelium, differentiation, cell reprogramming

## Abstract

Failure of corneal endothelium cell monolayer is the main cause leading to corneal transplantation. Autologous cell-based therapies are required to reconstruct *in vitro* the cell monolayer. Several strategies have been proposed using embryonic stem cells and induced pluripotent stem cells, although their use has ethical issues as well as limited clinical applications. For this purpose, we propose the use of dental pulp stem cells isolated from the third molars to form the corneal endothelium cell monolayer. We hypothesize that using dental pulp stem cells that share an embryological origin with corneal endothelial cells, as they both arise from the neural crest, may allow a direct differentiation process avoiding the use of reprogramming techniques, such as induced pluripotent stem cells. In this work, we report a two-step differentiation protocol, where dental pulp stem cells are derived into neural crest stem-like cells and, then, into corneal endothelial-like cells. Initially, for the first-step we used an adhesion culture and compared two initial cell sources: a direct formation from dental pulp stem cells with the differentiation from induced pluripotent stem cells. Results showed significantly higher levels of early stage marker AP2 for the dental pulp stem cells compared to induced pluripotent stem cells. In order to provide a better environment for neural crest stem cells generation, we performed a suspension method, which induced the formation of neurospheres. Results showed that neurosphere formation obtained the peak of neural crest stem cell markers expression after 4 days, showing overexpression of AP2, Nestin, and p75 markers, confirming the formation of neural crest stem-like cells. Furthermore, pluripotent markers Oct4, Nanog, and Sox2 were as well-upregulated in suspension culture. Neurospheres were then directly cultured in corneal endothelial conditioned medium for the second differentiation into corneal endothelial-like cells. Results showed the conversion of dental pulp stem cells into polygonal-like cells expressing higher levels of ZO-1, ATP1A1, COL4A2, and COL8A2 markers, providing a proof of the conversion into corneal endothelial-like cells. Therefore, our findings demonstrate that patient-derived dental pulp stem cells may represent an autologous cell source for corneal endothelial therapies that avoids actual transplantation limitations as well as reprogramming techniques.

## Introduction

Corneal transplantation, known as keratoplasty, is the most frequently performed type of transplant worldwide, with over 185,000 procedures performed each year (Hatou and Shimmura, 2019). The main indication of corneal transplantation in the US is Fuchs dystrophy, a disease related to the innermost corneal layer, known as corneal endothelium (Gain et al., 2016). In fact, endothelial keratoplasties, where only the corneal endothelium is replaced, represent 60% of all corneal transplants, and this percentage is increasing every year (Eye Bank Association of America, 2018). The lack of donors together with an increased demand for corneal tissue due to population aging, urges to find alternative solutions (Hatou and Shimmura, 2019). In this context, cell-based therapies are being developed in order to overcome actual limitations (Hatou and Shimmura, 2019).

Considering that the corneal endothelium is the most replaced layer and that it consists of a monolayer of corneal endothelial cells (CEC), it is necessary to obtain such layered cell structure. Hence, the aim of these cell-based therapies is to restore damaged endothelium by conforming cells *in vitro* with similar characteristics to those of native cells, using in most cases, cells obtained from donors. Nevertheless, this remains a great challenge as CEC have a restricted proliferative ability in humans, limiting its feasibility (Joyce, 2003). Furthermore, donor corneas are generally from older patients and these cells present even lower proliferation rates, being more prone to senescence than younger corneas (Senoo and Joyce, 2000; Miyata et al., 2001; Joyce, 2012; Katikireddy et al., 2016). In order to overcome the senescence state of cells, a recent study cultured CEC from a young donor and injected them with ROCK inhibitor, which has been described to reduce intraocular pressure, to increase CEC cell density and to promote the typical polygonal monolayer phenotype (Okumura et al., 2016; Kinoshita et al., 2018). The expanded cultured CEC from the donor were successfully placed in the host patient achieving increased cell densities after 24 weeks, which was ascribed to the ROCK inhibitor (Kinoshita et al., 2018). Despite of the recent efforts, patient still require local or systemic immunosuppression to prevent corneal tissue rejection, which is the main cause of corneal transplant failure. Consequently, an alternative autologous CEC source is required.

In this sense, recent studies have shown the use of somatic cells from the same patient to produce induced pluripotent stem cells (iPSC) that can be differentiated into CEC (Chakrabarty et al., 2018). Promising results have been achieved with these cells, mainly consisting of a two-step process based on the natural CEC embryological development from the neural crest (NC). This process consists in a first-step, where cells are differentiated into NC stem cells (NCSC) followed by a second-step, where NCSC are differentiated into CEC (Wagoner et al., 2018; Brejchova et al., 2019). Despite of the positive results, their clinical application is hindered due to safety issues. Furthermore, their production requires a previous tedious step in which cells are reprogrammed from somatic cells, mainly fibroblasts, into cells with a pluripotent status, requiring the use of gene transfection and long and expensive culture periods.

Hence, we propose the use of patient-derived dental pulp stem cells (DPSC) as an alternative approach for corneal endothelial therapy. DPSC can be easily obtained and cultured from third molars using a non-invasive technique, which avoids ethical and safety issues (Achilleos and Trainor, 2012). Moreover, DPSC present high proliferation rates at young and older donors and are able to preserve its stemness properties for *in vitro* long times of culture (Bressan et al., 2012). DPSC exhibit a great differentiation potential and have been differentiated into many cell types (Zhang et al., 2008; Ferro et al., 2014). Interestingly, DPSC, as well as CEC, arise from cranial NC and present similar characteristics to NC progenitor or stem cells (Achilleos and Trainor, 2012; Lwigale, 2015; Pisciotta et al., 2018). Some recent studies proposed as well the use of DPSC as a possible cell source, although these works were mainly related with the corneal epithelium or the stroma (Syed-Picard et al., 2015; Kushnerev et al., 2016). Furthermore, DPSC are considered as low immunogenic cells and present immunomodulatory properties, allowing successful DPSC grafts, both autologous and heterologous (Zhao et al., 2012; Pisciotta et al., 2020). Therefore, in this work we aim to determine if patient-derived DPSC, which share the same embryological origin with CEC, can be used as a potential autologous source for corneal endothelial regeneration, which to the best of our knowledge, has not been previously proposed.

For doing this, we propose a two-step protocol, involving the dedifferentiation of DPSC into NCSC, followed by their subsequent differentiation into CEC. We hypothesize that the initial dedifferentiation process can overcome the limitations of the cellular reprogramming from initial somatic cells, mainly fibroblasts, into iPSC and their subsequent differentiation into NCSC, reducing cost, time and avoiding safety issues (Liu et al., 2011). In order to validate the hypothesis, iPSC will also be produced from DPSC, allowing the comparison of direct conversion (dedifferentiation) from DPSC into NCSC as well as the differentiation from reprogrammed iPSC.

## Materials and Methods

### Dental Pulp Stem Cells Isolation and Culture

Dental pulp was extracted for orthodontic reasons from human third molars of healthy patients and obtained with informed consent from donors. DPSC were isolated modifying our previous protocol (Núñez-Toldrà et al., 2017, 2019). Briefly, dental pulp was digested with 3 mg/mL collagenase I (Sigma-Aldrich) at 37°C for 60 min. Disaggregated pulps were washed with phosphate-buffered saline (PBS) (Sigma-Aldrich) and cultured in expansion medium at 37°C in a 5% CO_2_ incubator. Cell expansion medium consisted of Dulbecco's modified Eagle's medium (DMEM) high glucose (Thermo Fisher Scientific) supplemented with 10% fetal bovine serum (FBS, Sigma-Aldrich), 1% GlutaMAX and 1% penicillin-streptomycin (Thermo Fisher Scientific). When DPSC reached 70% confluence, cells were detached using TrypLE™ Express Enzyme (Thermo Fisher Scientific) and re-plated or used for experiments. Cells were used until a maximum passage of 4. These cells were previously characterized and showed high expression levels of CD29 (99.63%) and CD105 (92.15%) markers, and low expression levels of CD45 (0.04%), analyzed by FACS (Núñez-Toldrà et al., 2017). Furthermore, pluripotent markers OCT3/4 (76.72%) and NANOG (30.18%) were also expressed. Moreover, DPSC were analyzed by immunofluorescence, western blot and/or RT-PCR and were positive for the pluripotent markers Oct3/4, SSEA4, Nanog, and Sox 2 (Núñez-Toldrà et al., 2017).

This study has been performed with ethics approval by the Research Ethical Committee from the *Universitat Internacional de Catalunya*, under the study code BIO-ELB-2013-03.

### Production of Induced Pluripotent Stem Cells

#### Cell Reprogramming With Sendai Virus

In order to compare the direct dedifferentiation from DPSC into NCSC with the differentiation of iPSC into NCSC, we reprogrammed DPSC into iPSC. For the reprogramming process, DPSC were reprogrammed using the CytoTune™ Reprogramming System, a non-integrating viral vector (Medvedev et al., 2010). Briefly, cells were plated in a 6-well plate at a cell density of 1 × 10^5^ cells/cm^2^. After 48 h, the transduction of the Yamanaka factors (Oct4, Sox2, Klf4, and c-MYC) was performed at an infection multiplicity of 3 using Sendai virus. Twenty-four hours after the transduction, culture medium was replaced with fresh media to remove the reprogramming vectors. Then, culture media was changed every other day. Total RNA was extracted at day 21 of the reprogramming process for PCR analysis. The results were compared to day 0, which was considered as the negative control.

In order to verify the proper reprogramming of DPSC into iPSC, the same commercially available kit previously used, the CytoTune™ Reprogramming System, was followed, using human dermal fibroblasts, which were kindly donated by the Center for Applied Medical Research (CIMA, University of Navarra). Fibroblasts were used as these were the cells recommended as positive controls by the manufacturers of the commercially available kit. Fibroblasts were cultured in cell expansion medium at a cell density of 3 × 10^3^ cells/cm^2^. The medium was changed every 2–3 days and when cells reached 70% confluence they were used for reprogramming process.

#### Reprogrammed Cells in Feeder and Feeder-Free Conditions

One week after the transduction, the transduced cells were plated on feeder dishes, which provide an extracellular matrix needed for the maintenance of an undifferentiated state until the formation of iPSC colonies (Llames et al., 2015).

For the preparation of the feeder layer, mouse fibroblasts SNL 76/7 (Cell Biolabs) were prepared as indicated in the commercial protocol. Briefly, cells were cultured at a cell density of 2 × 10^4^ cells/cm^2^ in expansion medium adding 0.1 mM MEM non-essential aminoacids (Sigma-Aldrich). When cells reached 90% of confluence, they were inactivated using 10 μg/mL mitomycin C (Sigma-Aldrich) and resuspended in DMEM. After 2 h, cells were washed three times with 1X PBS, then detached and used for experiments.

After 24 h of culture in feeder layer, culture medium was changed to iPSC medium, which consisted of Knock-out DMEM/F-12 (Thermo Fisher Scientific), 20% Knockout Serum Replacement (Thermo Fisher Scientific), 0.1 mM MEM non-essential aminoacids, 0.1 mM 2-mercaptoethanol (Thermo Fisher Scientific), 4 ng/mL basic fibroblast growth factor (bFGF, Sigma-Aldrich), 1% GlutaMAX, and 1% penicillin-streptomycin.

Finally, after 3 weeks of culture in feeder dishes, iPSC were detached using collagenase type IV (Thermo Fisher Scientific) and were expanded for 4 more weeks. Then, 8 weeks after transduction, colonies were adapted to feeder-free conditions using 10 μg/mL vitronectin-coated dishes (STEMCELL Technologies).

### First Step Cell Differentiation

#### Neural Crest Stem Cells Production

The DPSC dedifferentiation into NCSC was considered as the crucial step for the formation of CEC precursors. We followed a modified differentiation process based on previous results, which consisted on culturing cells on adhesion conditions while using a differentiation medium (Menendez et al., 2013). In this adhesion method, DPSC dedifferentiation was compared with the differentiation of the previously described iPSC-derived from DPSC. Both DPSC and iPSC-derived from DPSC were incubated in NC induction medium at a cell density of 0.5 × 10^3^ cells/cm^2^ for 21 days. The NC induction medium consisted of DMEM-F12 (Thermo Fisher Scientific) supplemented with 1X B-27 supplement (Thermo Fisher Scientific), 1X N-2 supplement (Thermo Fisher Scientific), 20 ng/mL epidermal growth factor (EGF), 20 ng/mL bFGF, 5 ng/mL heparin (Sigma-Aldrich), and 2 mM L-alanyl-L-glutamine (Sigma-Aldrich). Medium was changed every 2–3 days. In order to analyze cell phenotype, total RNA was extracted at day 21. NCSC markers AP2 and p75 were analyzed. RNA results were compared to day 0, which corresponds to undifferentiated cells.

After initially comparing the traditional iPSC differentiation method with the dedifferentiation from DPSC, we then compared the adhesion culture with a suspension method. The suspension method is based on the formation of neurospheres (NS) (Kawano et al., 2017; Pisciotta et al., 2018; Hong and Do, 2019). For this purpose, DPSC were cultured on ultra-low adhesion 24-well plates (Corning) in the previously described NC induction medium at a cell density of 2 × 10^4^ cells/well. Cells were forced to aggregate among them, forming NS-like structures. The culture was monitored by visual inspection using an optical microscope (Olympus CKX41, Nikon). The optimum size was considered to be below 250 μm in order to avoid hypoxia. The culture finalized once the desired NS size was reached. Total RNA was extracted every day of dedifferentiation for PCR analysis and was compared to day 0, corresponding to undifferentiated DPSC cells. NCSC markers AP2, Nestin and p75, and pluripotent markers Oct4, Nanog, and Sox2 were analyzed. The diameter of NS was measured at days 1, 2, 3, 4, and 5 of suspension culture using ImageJ software (U.S. National Institutes of Health).

High cell density is needed in the second step of differentiation in order to mimic the natural conditions in which CEC are found. For this purpose, once the NS were obtained, these were cultured for 15 days in adhesion conditions to allow cell expansion. Total RNA was extracted when NS were formed (day 4) and at the end of the expansion process (day 19), in order to verify if the NCSC phenotype was maintained during the expansion process. RNA results were compared to day 0, which were undifferentiated DPSC cells. NCSC markers AP2, Nestin and p75, and pluripotent markers Oct4, Nanog, and Sox2 were analyzed.

### Second Step Cell Differentiation

#### Human Corneal Endothelial Cell Conditioned Medium Preparation

All assays were adhered to the Declaration of Helsinki. Six non-transplantable human corneoscleral rims were obtained from the Florida Lions Eye Bank (Miami, USA). Descemet's membrane and corneal endothelium were isolated as previously described with some modifications (Sabater et al., 2017). Briefly, corneal tissue was rinsed with PBS. The iris and possible contaminations were removed with tweezers. Subsequently, the Descemet's membrane with the corneal endothelium were gently removed with tweezers and cultured in CEC medium at 37°C in a 5% CO_2_ incubator. CEC medium consisted of DMEM high glucose (Thermo Fisher Scientific) supplemented with 5% FBS, 1% GlutaMAX, 2 ng/mL bFGF, 0.1 mM 2-mercaptoethanol, 10 ng/mL heregulin beta (Sigma-Aldrich), 10 ng/mL activin A (Miltenyi Biotec), 200 ng/mL IGF-I (Sigma-Aldrich), and 1% penicillin-streptomycin. Previous reports have demonstrated that conditioned medium enhanced cell differentiation (Chen et al., 2015, 2018). Therefore, culture medium was replaced every 2 days, and was collected once CEC reached confluence. Collected medium (conditioned medium), was filtered (0.45 μm) and stored at −80°C in order to maintain its biological activity. Conditioned medium was used in the second step of cell differentiation.

#### Corneal Endothelial Cell Differentiation

Once the proper NCSC number was reached, the conditioned medium from isolated human CEC was used to induce the differentiation into CEC. For this purpose, NCSC-derived from DPSC were cultured at a cell density of 2 × 10^5^ cells/cm^2^ for 15 days in conditioned medium. Conditioned medium was changed every 2 days. Total RNA was extracted at days 12 and 19 of differentiation for PCR analysis. CEC markers Zona Occludens-1 (ZO-1), ATP1A1, collagen type IV and VIII (COL4A2 and COL8A2) were analyzed. As a positive control, RNA extracted from isolated CEC was used. Results were compared to day 0, which corresponded to undifferentiated DPSC cells.

**Figure 1:**
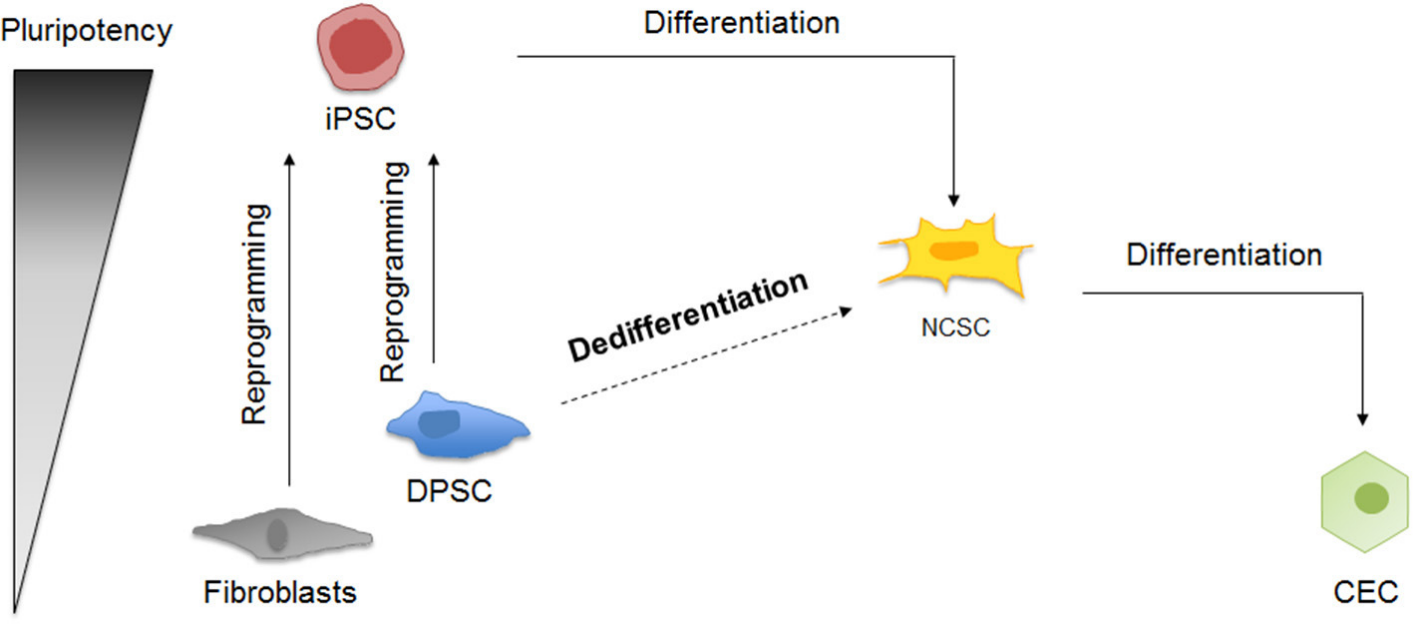
Schematic representation of corneal endothelial differentiation based on the pluripotency levels. On the one hand, somatic cells as fibroblasts and DPSC can be reprogrammed into iPSC, which can be differentiated into NCSC and then into CEC. On the other hand, DPSC could be dedifferentiated into NCSC, and then differentiated into CEC.

The different cellular processes taking place in this study are summarized in Figure 1.

### Cell Morphology Analysis

As an initial characterization, cellular morphology was analyzed. For this purpose, samples were fixed using 4% paraformaldehyde (Sigma-Aldrich) for 20 min at room temperature. Then, cells were washed with PBS and were observed by optical microscopy (Olympus CKX41, Nikon) to obtain an overall cell morphology.

### Quantitative Real-Time Reverse Transcription Polymerase Chain Reaction (qRT-PCR)

The relative expression of characteristic genes was analyzed to verify the differentiation process by quantitative real time – polymerase chain reaction (qRT-PCR). Total RNA was isolated using NucleoSpin RNA kit (Macherey-Nagel) and reversed transcribed into cDNA using Transcriptor First Strand cDNA Synthesis Kit (Roche) following the manufacturer's indications. Specific primers, shown in Table 1, and FastStart Universal SYBR Green Master (Roche) were used to amplify the cDNA. Lastly, a CFX96 real-time PCR Detection System (Bio-Rad) was used for the quantitative real-time PCR process.

**Table 1:** List of primers used for cDNA amplification in quantitative RT-PCR.

**Gene**	**Primer**	**Sequence (5^**′**^-3^**′**^)**	**Accession number**
Oct4	Forward	AGCGAACCAGTATCGAGAAC	NM_002701
	Reverse	TTACAGAACCACACTCGGAC	
Nanog	Forward	TGAACCTCAGCTACAAACAG	NM_024865
	Reverse	TGGTGGTAGGAAGAGTAAAG	
Sox2	Forward	GCCGAGTGGAAACTTTTGTCG	NM_003106
	Reverse	GGCAGCGTGTACTTATCCTTCT	
p75	Forward	CCTACGGCTACTACCAGGATG	NM_002507
	Reverse	CACACGGTGTTCTGCTTGT	
AP2	Forward	AGGTCAATCTCCCTACACGAG	NM_003220
	Reverse	GGAGTAAGGATCTTGCGACTGG	
Nestin	Forward	CTGCTACCCTTGAGACACCTG	NM_006617
	Reverse	GGGCTCTGATCTCTGCATCTAC	
ATP1A1	Forward	TGTGATTCTGGCTGAGAACG	NM_001160234
	Reverse	TGCTCATAGGTCCACTGCTG	
COL4A2	Forward	TAAGGGTGAAAAGGGTGACG	NM_001846
	Reverse	ACTGATCTGGGTGGAAGGTG	
COL8A2	Forward	GAACTGGACGGTTCTGTGGT	NM_003759
	Reverse	AATTGCCGAAAGAGCTTGAA	
ZO-1	Forward	AGTTTGGCAGCAAGAGATGG	NM_001355015
	Reverse	GCTGTCAGAAAGATCAGGGA	
GAPDH	Forward	CTGGTAAAGTGGATATTGTTGCCAT	NM_002046
	Reverse	TGGAATCATATTGGAACATGTAAACC	
b-ACTIN	Forward	AGAGCTACGAGCTGCCTGAC	NM_001101
	Reverse	AGCACTGTGTTGGCGTACAG	

### Immunofluorescence Assay

In order to analyze protein expression of iPSC derived from DPSC, the Pluripotent Stem Cell 4-Marker Immunocytochemistry Kit protocol (Invitrogen) was performed. Briefly, cells were fixed with 4% paraformaldehyde (Invitrogen) for 15 min at room temperature. Later, cells were permeabilized with 1% Saponin at room temperature for 15 min. After this stage, cells were blocked with 3%BSA for 30 min at room temperature. After two washes with PBS, the primary antibodies (Supplementary Table 1) were added and incubated for 3 h, at 4°C in the dark. Samples were then washed three times with PBS. Secondary antibody (Supplementary Table 1) was added and incubated for 1 h at room temperature in the dark. After two washes, cells were then counterstained with 1 μg/mL 4′,6-diamidino-2-phenylindole (DAPI) for 5 min and then rinse twice with DPBS. Then, samples were observed using an inverted fluorescence microscope with an epifluorescence attachment (Eclipse TS100; Nikon).

For the case of the NS, an initial step was performed before the immunofluorescence staining. For this reason, NS were firstly embedded with O.C.T. Compound (Sakura) and were frozen at −20°C overnight and then at −80°C for at least 24 h. Later, samples were cryosectioned (Cryotome SME, Thermo) and kept on super frost slides. The immunofluorescence assay was performed as described in the previous paragraph.

### Statistical Analysis

All experiments were performed in technical triplicates and repeated at least in two independent experiments.

Mann-Whitney *U*-test was used to analyze significant differences. Differences with a *P*-value of < 0.05 were considered statistically significant.

GraphPad Prism version 6 (GraphPad Software Inc.) was used to represent the quantitative data presented as mean and standard deviation (SD). Statistical analyses were carried out using Statistical Package for the Social Sciences (SPSS) 21 (IBM).

## Results

### Production and Characterization of Induced Pluripotent Stem Cells

**Figure 2:**
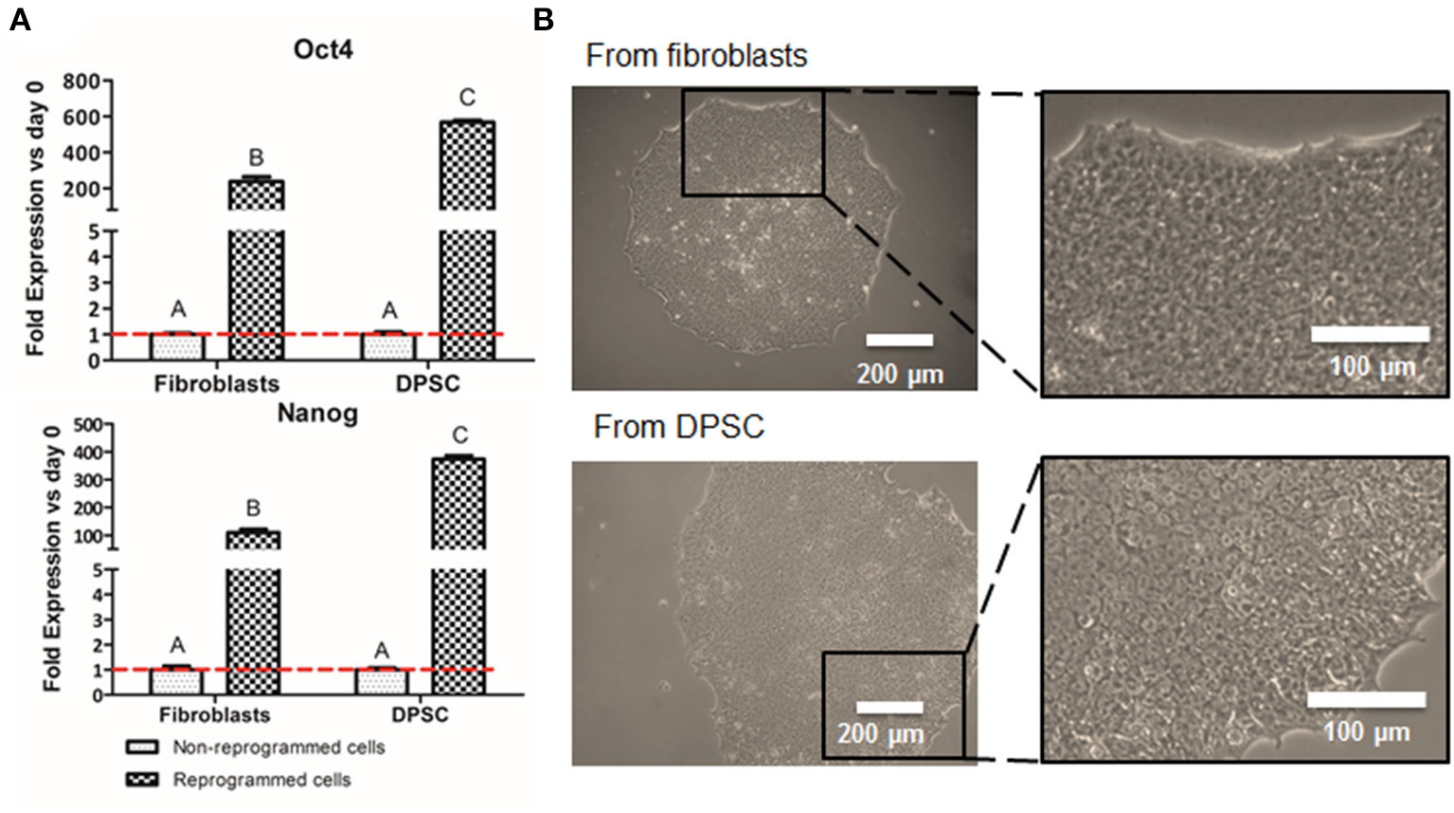
Cell reprogramming from DPSC and fibroblasts. Relative mRNA expression in DPSC and fibroblasts (positive control), before and after cell reprogramming **(A)**. Pluripotent expression of Oct 4 and Nanog by quantitative RT-PCR. Optical microscope images of iPSC colonies cultured in VTN-coated dishes from fibroblasts (positive control) and from DPSC **(B)**. *Scale bars: 200 μm (lower magnification) and 100 μm (higher magnification). **Data presented are means ± SD, *n* = 3 technical replicates per donor and experimental group. Different letter denotes significant differences (*p* < 0.05). Same letter denotes non-significant differences.

The production of iPSC was performed to analyze whether the pluripotent-like status is required for the production of NCSC or a direct conversion from DPSC into NCSC is a plausible route. These iPSC were obtained from DPSC to allow the comparison with the direct conversion of DPSC into NCSC, whereas the well-established fibroblasts, were used as positive control for the reprogramming process. After 2 weeks of the transduction, first emerging iPSC colonies started to appear, observing how cells started to change its spindle-like morphology into a more rounded morphology. In order to validate that the cells were correctly reprogrammed, pluripotent expression markers were evaluated. Results showed that cells reprogrammed from DPSC presented statistically significant higher levels of gene expression of the pluripotent markers Oct4 and Nanog compared to the non-reprogrammed cells as well as to the positive control (Figure 2A). Furthermore, iPSC-derived from DPSC were also analyzed by immunofluorescence and cells were positive for the pluripotent markers Oct4, SSEA4, TRA-1-60, and Sox2 (Supplementary Figure 1).

Colonies were carefully picked at week 4 post-transduction and were expanded in feeder conditions for at least 4 more weeks. Adaptation to feeder-free conditions, on vitronectin-coated dishes, was performed 8 weeks after transduction. iPSC on vitronectin-coated dishes presented pluripotent-like characteristics such as the formation of cobblestone-like colonies, presenting individual cells with a high nuclear to cytoplasm ratio (Figure 2B).

### Cell Differentiation

#### Neural Crest Stem Cells Production

**Figure 3:**
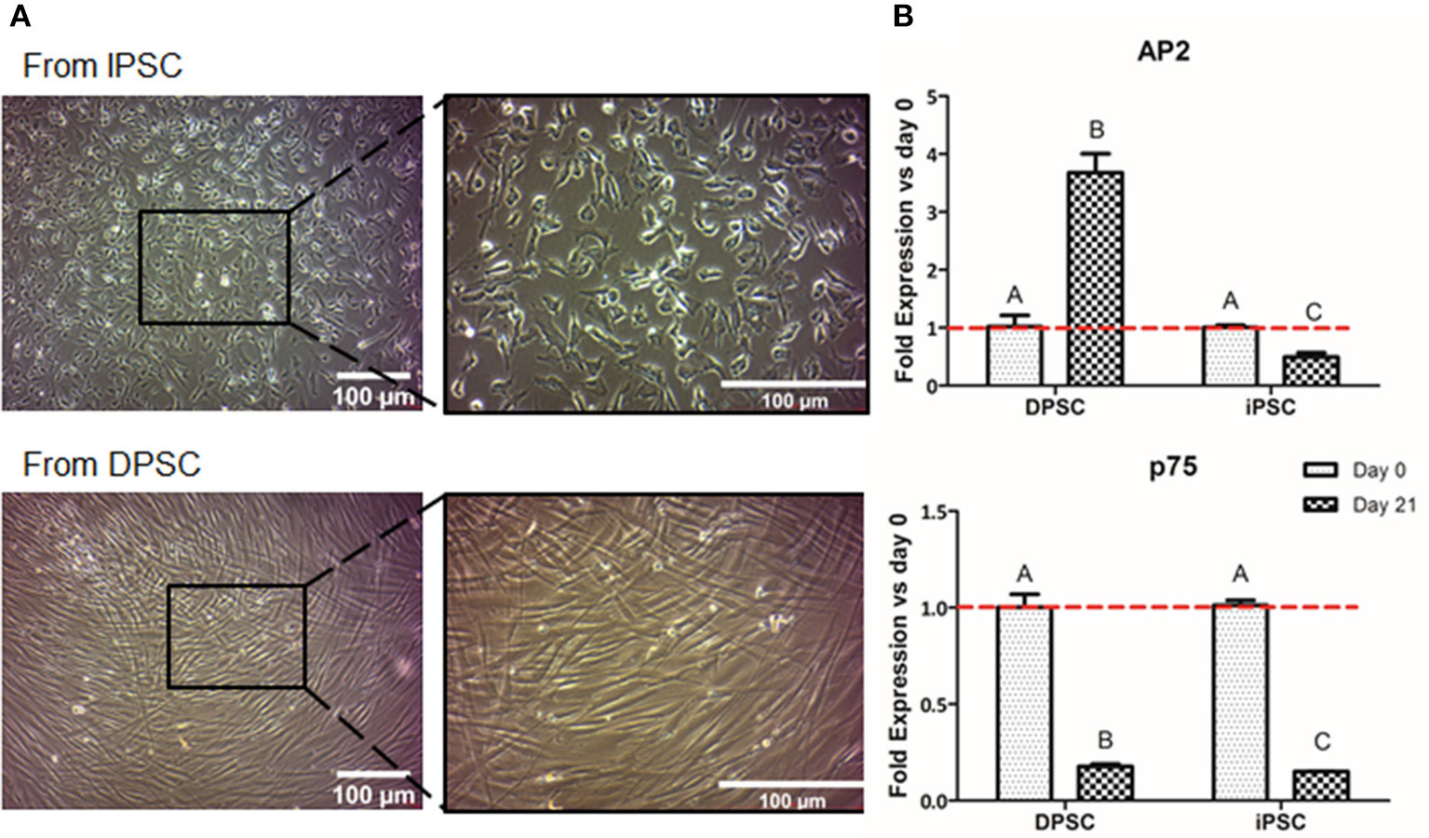
Formation of NCSC using an adherent method. Microscope images of NCSC-derived from iPSC and from DPSC at day 21 of differentiation **(A)**. Relative mRNA expression in NCSC-derived from DPSC and iPSC, at days 0 and 21, analyzed by quantitative RT-PCR **(B)**. *Scale bar: 100 μm. **Data presented are means ± SD, *n* = 3 technical replicates per experimental group. Different letter denotes significant differences (*p* < 0.05). Same letter denotes non-significant differences.

We first evaluated the formation of NCSC using adherent conditions. Regarding the overall cell morphology, cells presented a more stellate typical NCSC morphology at day 21 of differentiation when the differentiation was performed from iPSC (Figure 3A). In contrast, dedifferentiated cells from DPSC exhibited a more elongated morphology after 21 days of dedifferentiation (Figure 3A).

Aiming to analyze the fold gene expression, in the case of NCSC-derived from iPSC, the NCSC markers AP2 and p75 decreased throughout the differentiation process (Figure 3B). However, in the case of NCSC-derived directly from DPSC, dedifferentiated cells presented statistically significant higher levels of the early NCSC marker AP2, whereas the mid-term marker p75 was down-regulated at day 21 of the dedifferentiation (Figure 3B).

**Figure 4:**
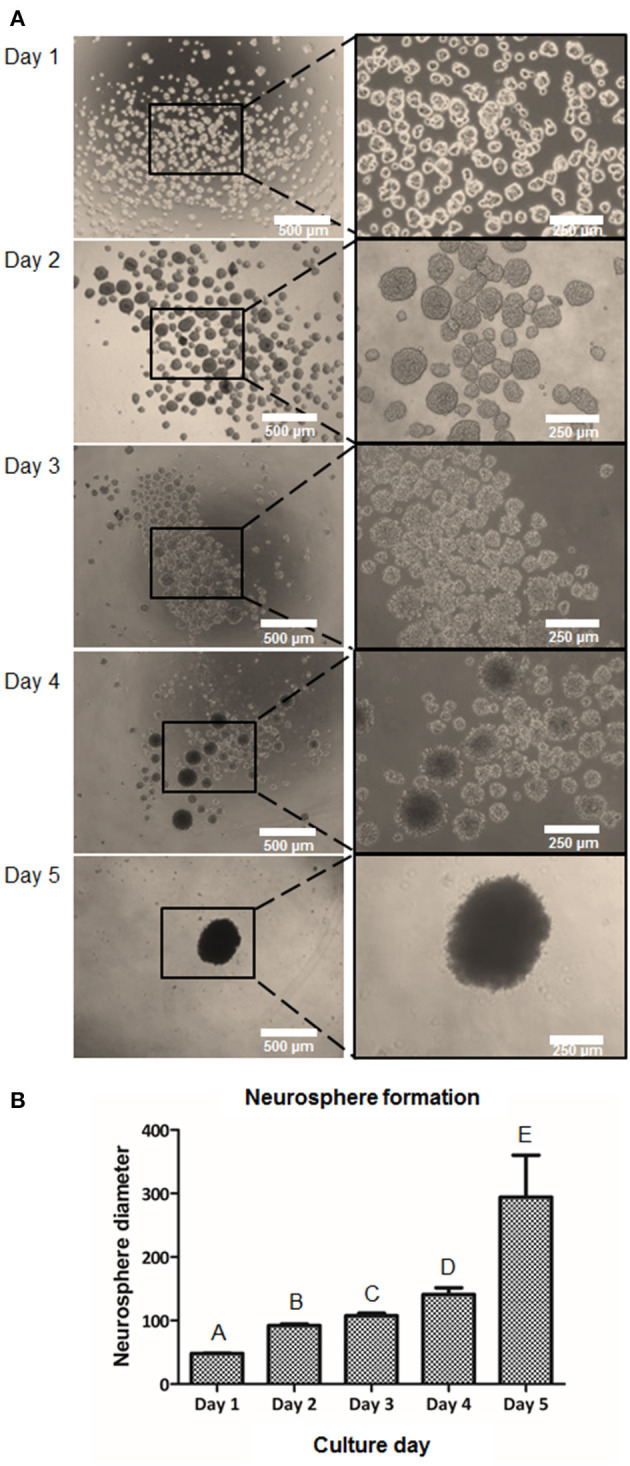
Formation of NCSC from DPSC using a suspension method. Microscope images of cell morphology at days 1, 2, 3, 4, and 5 of NCSC formation in suspension culture **(A)**. Neurosphere diameter at days 1–5 of suspension culture **(B)**. *Scale bars: 500 μm (lower magnification) and 250 μm (higher magnification). **Data presented are means ± SD. Different letter denotes significant differences (*p* < 0.05). Same letter denotes non-significant differences.

In order to induce a higher cell-to-cell interaction, the suspension culture was performed. Ideally, the cell-to-cell contact could help mediate in higher NCSC induction capability. Therefore, we cultured DPSC for 5 days in suspension culture plates. Floating clusters or cell aggregates started to appear at day 1 and their diameter increased until day 5 (Figure 4A). As excessive NS diameters were hypothesized to induce hypoxia, limiting as well the diffusion of nutrients in the center of the NS, its diameter was monitored to prevent excessive NS sizes. As shown in Figure 4B, at day 1, cell aggregates presented values of NS below 70 μm. The NS size progressively increased during the next days and presented an average diameter of 100–150 μm at days 2, 3, and 4. At day 5 of culture, the number of NS present in the culture abruptly decreased presenting a significant number of floating cells in the culture. Moreover, the diameter of the NS at day 5 was excessively big, being around 300 μm (Figure 4B).

**Figure 5:**
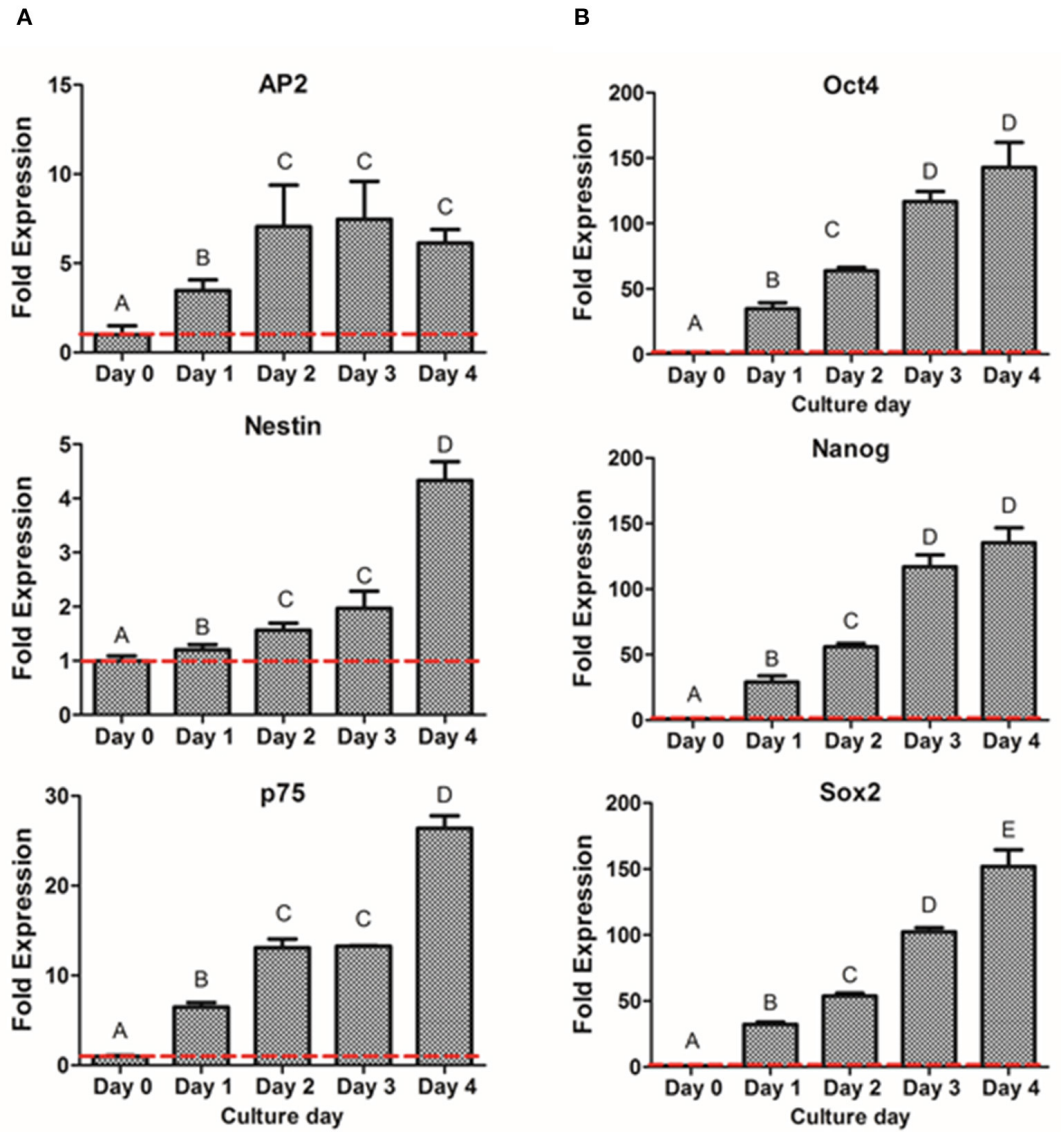
Gene expression of NCSC formation from DPSC in suspension culture. Relative mRNA expression of NCSC markers AP2, Nestin, and p75 **(A)** and pluripotent markers Oct4, Nanog, and Sox2 **(B)** analyzed by quantitative RT-PCR. *Data presented are means ± SD, *n* = 3 technical replicates. Different letter denotes significant differences (*p* < 0.05). Same letter denotes non-significant differences.

To further characterize the dedifferentiation process, fold gene expression was evaluated in order to obtain the optimal peak day of the process. The analysis of neural crest related markers (Figure 5A) showed that the early NCSC transcription factor AP2 exhibited its peak level at day 3, not presenting statistically significant differences with day 2 and day 4. However, the NCSC markers Nestin and p75, presented the highest expression levels at day 4, presenting significant differences with the other culture days. Interestingly, pluripotent markers Oct4, Nanog, and Sox2 showed as well a significant increase with time, showing its peak level at day 4 of dedifferentiation (Figure 5B). Moreover, NS at day 4 were also analyzed by immunofluorescence assay for the expression of p75 and results confirmed its expression at the protein level (Supplementary Figure 2).

**Figure 6:**
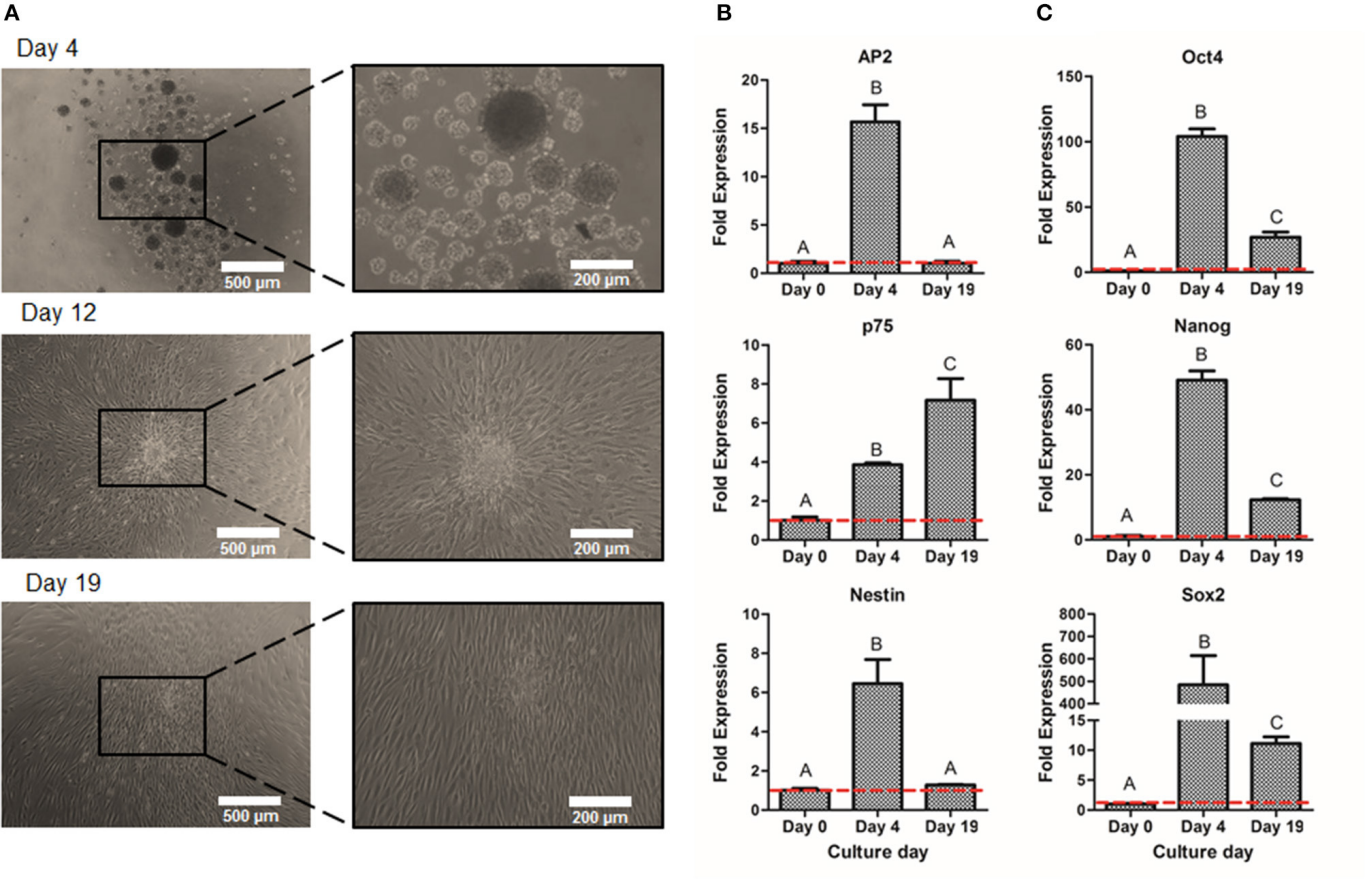
Expansion of NCSC from DPSC. Microscope images of NCSC-derived from DPSC at days 4, 12, and 19 **(A)**. Relative mRNA expression in NCSC-derived from DPSC, at days 0, 4, and 19 of NCSC markers AP2, Nestin, and p75 **(B)** and of pluripotent markers Oct4, Nanog, and Sox2 **(C)**, by quantitative RT-PCR. *Scale bars: 500 μm (lower magnification) and 200 μm (higher magnification). **Data presented are means ± SD, *n* = 3 technical replicates. Significant differences compared to day 0 (*p* < 0.05).

Once the optimal peak day of NS formation was determined, NS were then cultured on adherent conditions for 15 days more in order to obtain larger amounts of NCSC. As shown in Figure 6A, cells migrated from the NS, allowing the diffusion of cells from the original NS, hence disrupting its morphology. These migrated NC cells, reached confluence after 15 days of expansion (Figure 6A). In order to confirm that NC migrating cells were able to maintain NCSC phenotype, gene expression levels were analyzed (Figure 6B). The NCSC markers AP2 and Nestin showed an initial up-regulation (during the 4 initial days of neurosphere formation) followed by a down-regulation at day 19. Both levels decreased to values similar to those at day 0, which corresponds to undifferentiated DPSC (Figure 6B). In contrast, the expression of the mid-term NCSC marker p75 presented an increase expression throughout the expansion process, obtaining its highest expression at day 19 (Figure 6B). As shown in Figure 6C, pluripotent markers Oct4, Nanog, and Sox2 followed a similar trend to AP2 and Nestin, which were significantly up-regulated during the initial suspension culture (day 4) while its expression was down-regulated at the end of the expansion culture (day 19).

#### Corneal Endothelial Cell Differentiation

**Figure 7:**
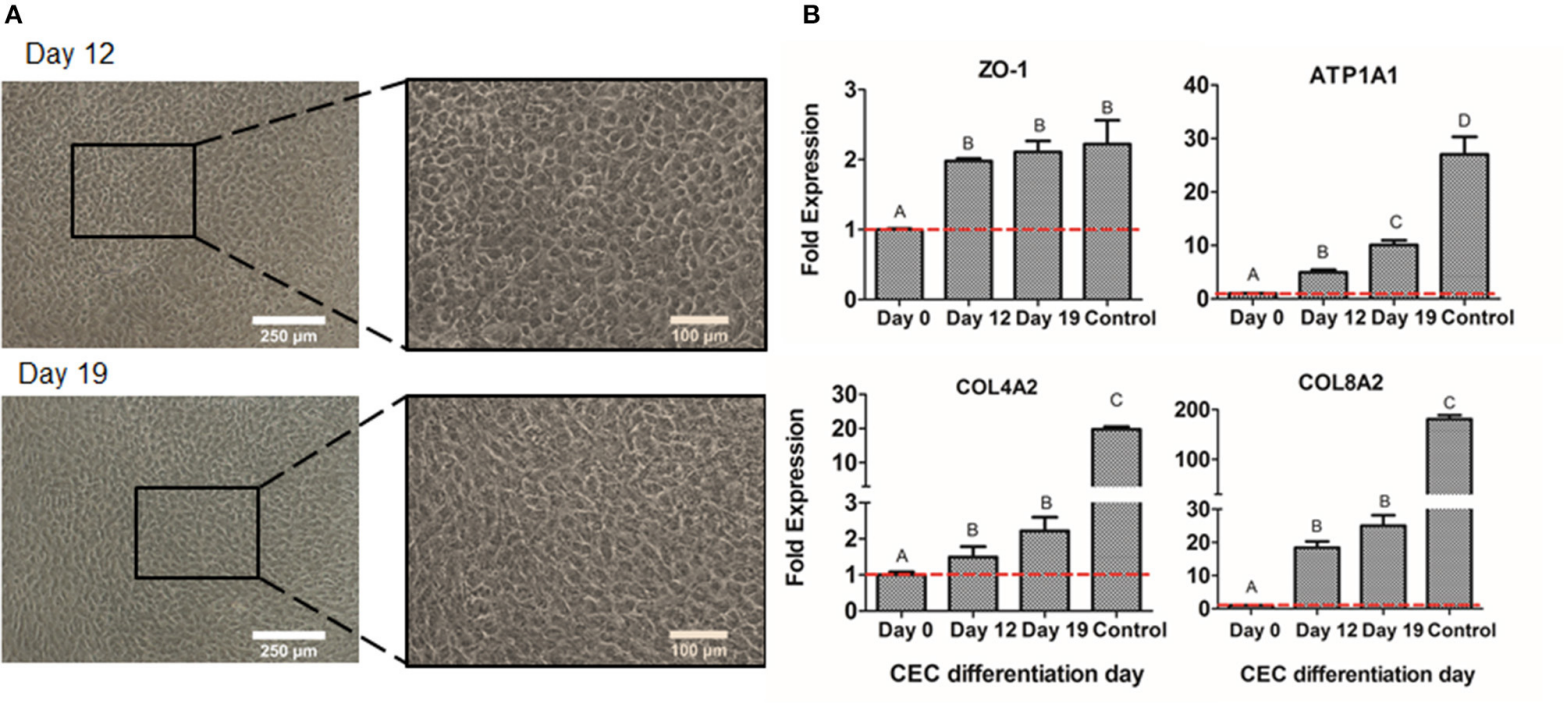
CEC differentiation from DPSC. Microscope images of CEC-derived from DPSC at days 12 and 19 **(A)**. Relative mRNA expression in CEC-derived from DPSC, at days 0, 4, and 19 of CEC markers ZO-1, ATP1A1, COL4A2, and COL8A2, by quantitative RT-PCR **(B)**. *Scale bars: 250 μm (lower magnification) and 100 μm (higher magnification). **Data presented are means ± SD, *n* = 3 technical replicates per experimental group. Different letter denotes significant differences (*p* < 0.05). Same letter denotes non-significant differences.

The second step of differentiation was to culture NCSC-derived from DPSC, corresponding to day 4 of dedifferentiation, in corneal endothelial conditioned medium for 15 days more. Cell morphology was observed with optical microscopy, showing that cells started to exhibit a polygonal morphology characteristic of CEC at day 12 of differentiation and this was maintained until the end of the differentiation process, corresponding to day 19 (Figure 7A).

Finally, we compared the values of different CEC markers during the differentiation process. As shown in Figure 7B, the gene expression of the typical CEC marker ZO-1 showed a significant increase at days 12 and 19 of the differentiation. Moreover, the Na^+^/K^+^-ATPase pump ATP1A1 expression showed a significantly progressive increase at days 12 and 19 (Figure 7B). Lastly, extracellular matrix components as COL4A2 and COL8A2 significantly increased at days 12 and 19 of differentiation compared to day 0, which are undifferentiated cells (Figure 7B). Although ZO-1 exhibited similar values to the control sample, which were freshly isolated CEC from donor corneas, the other markers presented decreased levels of gene expression (Figure 7B).

## Discussion

New cell therapies are needed for corneal endothelial regeneration in order to overcome problems related to corneal transplantation, which is the gold standard, such as global scarcity of healthy donor corneas or corneal tissue rejection (Gain et al., 2016). Dental pulp represents a very accessible source, as it contains stem cells with similar characteristics to NCSC due to their NC origin (Achilleos and Trainor, 2012). In the same way, CEC also derive from the NC (Lwigale, 2015). Therefore, in the present study, we have evaluated the feasibility of obtaining CEC from patient-derived DPSC, which could be used to treat corneal endothelial dysfunction using a safe and accessible source of autologous cells.

Initially, we evaluated the possibility of performing a direct differentiation from DPSC into CEC. This was based on previous work that showed the possibility to directly differentiate MSC or DPSC into endothelial cells, both *in vitro* and *in vivo* (Luzuriaga et al., 2019). In a similar way, we previously showed the ability of MSC to express endothelial markers when placed under an angiogenic stimuli (Perez et al., 2015). Nevertheless, the success of these previous works was based on the differentiation of MSC or DPSC into vascular endothelial cells rather than corneal endothelial cells. In the present work, despite it was hypothesized as a possible differentiation route, it was rapidly discarded as DPSC did not survive the corneal endothelial culture media.

Based on previous protocols and to the inability to have a direct conversion from DPSC to CEC (McCabe et al., 2015; Song et al., 2016), our differentiation process consisted of a two-step differentiation protocol, where DPSC were dedifferentiated into NCSC and, finally, into CEC. As the gold standard differentiation is the use of pluripotent stem cells as the cell source, we initially reprogrammed DPSC into iPSC in order to compare and validate the differentiation process of both cell types. Despite iPSC are a promising source for cell therapy, its limited clinical applicability due to safety issues, mainly regarding teratoma formation, opens the use of new alternatives to overcome this limitation. Research on iPSC is continuously growing, providing new alternative strategies to overcome the risk of teratoma formation. For instance, chemical treatment, genetic treatment, and immunological treatment are being studied to avoid the teratoma formation (Wuputra et al., 2020). In fact, the use of some small molecules, such as an inhibitor of stearoyl-CoA desaturase 1 (SCD1) or N-benzylnonanamide JC101, has been demonstrated to prevent teratoma formation (Ben-David et al., 2013a; Richards et al., 2014). Other studies have analyzed the prevention of teratoma formation with genetic treatment like the introduction of a suppressant of an antiapoptotic factor (Blum et al., 2009; Bedel et al., 2017). Moreover, prevention of teratoma formation has been also evaluated by immunological treatment, using antibodies against different antigens such as SSEA-5, claudin-6, or using cytotoxic antibodies (Tang et al., 2011; Ben-David et al., 2013b; Wyles et al., 2014). However, the use of iPS cells require long culture periods and high cost and, therefore, in the specific case of CEC, due to their common embryological origin with DPSC, we have chosen an alternative differentiation route that does not involve the use of iPSC.

Interestingly, our preliminary results showed that the gene expression of the pluripotent markers Oct4 and Nanog in reprogrammed cells derived from DPSC presented significantly higher levels than the positive control, which were fibroblasts (Figure 2A). This difference could be due to the later development in time of the DPSC, which are isolated from third molars, compared to fibroblasts, which are differentiated cells. In fact, DPSC endogenously express some pluripotent markers (Janebodin et al., 2011; Núñez-Toldrà et al., 2017), which has been described to enhance the reprogramming process (Kim et al., 2009; Sun et al., 2009; Chen et al., 2014). Therefore, the generation of iPSC seems to be easily performed and with higher pluripotent expression when using DPSC compared to fibroblasts. Furthermore, reprogrammed cells exhibited its typical cobblestone morphology with an elevated nuclear to cytoplasm ratio (Figure 2B), together with the expression of different pluripotent markers at the protein level (Supplementary Figure 1) as described by other groups (Zapata-Linares et al., 2016; Zhang et al., 2017).

Once iPSC-derived from DPSC were successfully generated, we then compared the conversion potential of iPSC and DPSC into NCSC. It has been described that aging reduces the regenerative potential of DPSC (Iezzi et al., 2019). In such cases, an initial pre-selection of cells with higher stem cells expression could be necessary in order to increase the differentiation efficiency. It was previously reported that iPSC could be differentiated into NCSC using an adherent culture (Menendez et al., 2013). Therefore, we used this strategy as a first approach for the formation of NCSC. Optical microscope images (Figure 3A) showed that the differentiation from iPSC exhibited cells with a stellate morphology typical for NCSC, similar to previous reports (Bajpai et al., 2010; Kawano et al., 2017), while the dedifferentiated cells directly from DPSC presented a more elongated shape (Figure 3A). Surprisingly, in the dedifferentiation process from DPSC into NCSC, the gene expression of typical NCSC markers showed that AP2 presented a significant up-regulation compared to the NCSC obtained from iPSC (Figure 3B). This could be related with the fact that undifferentiated DPSC already express NCSC markers, as previously demonstrated (Janebodin et al., 2011). For this reason, the formation of NCSC may be accelerated using DPSC compared to iPSC, which may need longer time of NC induction conditions for a complete differentiation. As DPSC seem to successfully generate NCSC, and in order to reduce time and costs, our next strategy was only performed using DPSC. Furthermore, iPSC were not used as control for the final differentiation into CEC as the duration of the differentiation processes from iPSC and DPSC are significantly different, making difficult the comparison between the obtained results.

We then evaluated if a suspension culture, which mimics the embryological process of gastrulation and promotes cell-to-cell interactions (Baker, 2008), could further enhance the formation of NCSC. As shown in Figure 4A, this system promoted the formation of cell aggregates at day 1. However, these cell aggregates that measured around 50 μm of diameter were not considered NS as its sizes normally range from 100 to 200 μm, as was previously reported (Xiong et al., 2011). NS between these sizes were already formed at day 2 of culture (Figure 4B) and their size progressively increased during the following days of culture. However, at day 5 of culture, NS started to disrupt its morphology and cell survival decreased, which was probably ascribed to a lower nutrient and oxygen diffusion into the NS inducing cell death. This is in accordance with a previous report which concluded that distances above 250 μm to culture medium may significantly limit the diffusion of nutrients and oxygen (Xiong et al., 2011). Therefore, gene expression was evaluated from day 0 (undifferentiated cells) until day 4 in order to confirm the formation of NCSC (Figure 5). Interestingly, the expression of the NCSC marker AP2 exhibited its peak before day 4, while the expression of Nestin and p75 presented its higher fold expression at day 4 of dedifferentiation (Figure 5A), also confirmed by p75 protein expression (Supplementary Figure 2). These results are in line with a previous report that showed that culturing DPSC in a suspension system preserved stemness and maintained the expression of neural crest markers (Pisciotta et al., 2018). Moreover, results were in accordance with our initial hypotheses, as AP2 is a transcription factor that is expressed as an early inductive signal, whereas p75 is a mid-late marker expressed in the migratory NC (Bajpai et al., 2010; Green et al., 2015). Furthermore, the gene expression of pluripotent markers such as Oct4, Nanog, and Sox2 (Figure 5B) showed an increasing expression throughout the different days of culture. This can be related with enhanced cell-to-cell interactions in the suspension conditions which could stimulate the beginning of gastrulation, which occurs in the earlier weeks of embryogenesis and hence presents the highest levels of pluripotency (Baker, 2008). Based on these results, we selected the suspension method for the formation of NCSC.

Prior to the formation of CEC (second-step of differentiation), which requires an initial high cell density, we expanded the NS in adherent conditions. As deducted from the suspension culture, optimum culture time was established to be 4 days. In this sense, it was important to verify if culturing the NS in adhesion after their formation could provide enough number of cells with adequate phenotype. Our results showed (Figure 6) that the expression of NCSC markers, AP2 and Nestin, and pluripotent markers decreased during the adherent expansion (day 19). These results are in line with a previous study that determined that pluripotent markers were inherently decreased in adherent culture (Elkabetz et al., 2008), which is related to the fact that the cell-to-matrix contact increased while the cell-to-cell interaction decreased. However, the expression of the NCSC marker p75 increased during the adherent expansion culture (Figure 6B). It has been described that p75 is related to migratory NC cells (Bajpai et al., 2010) and, therefore, it is expected to present higher levels during the expansion culture. Therefore, we determined that NCSC expansion using an adherent culture was not viable due to the altered phenotype and hence the optimum culture was to increase the initial number of DPSC in culture to obtain a sufficient number of cells using the suspension method for the subsequent differentiation.

Finally, once the first-step of the differentiation was achieved and confirmed, NCSC-derived from DPSC were differentiated into CEC. Other groups have determined that the culture with conditioned medium promotes the formation of CEC (Chen et al., 2015; Shen et al., 2017), so the use of conditioned medium from freshly isolated CEC was evaluated to induce the differentiation of NCSC-derived from DPSC. Optical microscope images showed that cells started to exhibit its typical polygonal morphology at day 12 of differentiation (Figure 7A), indicating that the differentiation process had already started, and was maintained until day 19. We then characterized the differentiated cells by gene expression, using characteristic CEC markers ZO-1, ATP1A1, COL4A2, and COL8A2 (Lachaud et al., 2014). As shown in Figure 7B, we identified higher levels of expression at days 12 and 19 of CEC differentiation of the tight junction ZO-1, the pump function ATP1A1, and the extracellular collagens COL4A2 and COL8A2, compared to undifferentiated DPSC, indicating that cells were differentiating into a CEC-like phenotype. Although the gene expression was higher in CEC-like cells derived from DPSC, its value was lower than the expression of human CEC, used as control. This is in line with a previous study which presented lower levels of gene expression of CEC-derived from ESC compared to human CEC or CEC cell line (McCabe et al., 2015; Yamashita et al., 2018).

In summary, our results have shown that patient-derived DPSC can be potentially dedifferentiated into NCSC using a short-time suspension culture, through the formation of NS as shown by our gene expression analysis. Furthermore, NCSC-derived from DPSC cultured in conditioned medium were able to generate CEC-like cells, which shared some similarities with native CEC such as polygonal morphology and gene expression of typical markers at the end of the differentiation. To our knowledge, no previous studied have proposed similar strategy, which may reduce the cost as well as increase the clinically ability to translate the results into clinical scenarios. The generation of patient-derived CEC would potentially overcome actual problems of corneal endothelial therapies. Despite the results shown are encouraging for the treatment of corneal endothelial cell therapy, further validation by analyzing the expression of the key markers at the protein level are needed. Furthermore, the differentiation process does not mimic the native environment of CEC such as the natural curvature of the cornea, the *in vivo* cross talks or the specific extracellular matrix composition. Further experiments mimicking these native conditions should enhance the expression of CEC-like cells into levels of expression similar to isolated CEC.

## Data Availability Statement

The raw data supporting the conclusions of this article will be made available by the authors, without undue reservation.

## Ethics Statement

The studies involving human participants were reviewed and approved by Research Ethical Committee from the Universitat Internacional de Catalunya, under the study code BIO-ELB-2013-03. Written informed consent to participate in this study was provided by the participants' legal guardian/next of kin.

## Author Contributions

BB and RN-T carried out the isolation and cell culture of stem cells. BB performed the cell reprogramming, cell differentiation, and its analysis. ES and AS performed the corneal endothelial cell culture. FG and RP participated in the design of the study, participated in the manuscript writing, and helped to draft the manuscript. All authors read and approved the final manuscript.

## Conflict of Interest

The authors declare that the research was conducted in the absence of any commercial or financial relationships that could be construed as a potential conflict of interest.

## Supplementary Material

Supplementary Figure 1 Protein analysis of iPSC-derived from DPSC. Immunofluorescence stained images for reprogrammed cells from DPSC. Cells were positive for the pluripotent markers Oct4 (A-red), SSEA4 (A-green), TRA-1-60 (B-red), and Sox2 (B-green). Blue color corresponds to nucleus (DAPI). Scale bars: 100 μm.

Supplementary Figure 2 Protein analysis of NCSC-derived from DPSC at day 4 of culture. Immunofluorescence stained images for dedifferentiated cells from DPSC. Neurospheres cells were positive for the NCSC markers p75. Blue color corresponds to nucleus (DAPI). Scale bar: 100 μm.

## Abbreviations

CEC: corneal endothelial cells
DPSC: dental pulp stem cells
IPSC: induced pluripotent stem cells
NC: neural crest
NCSC: neural crest stem cells
NS: neurospheres.
