# Supplementary material for: Discovering the Potential of Dental Pulp Stem Cells for Corneal Endothelial Cell Production: A Proof of Concept
Source: Front Bioeng Biotechnol. 2021 Jan 28;9:617724. doi: 10.3389/fbioe.2021.617724 (PMC7876244; doi:10.3389/fbioe.2021.617724)
Supplement: Supplementary file 1 [file Table_1.docx]

Table S1. List of antibodies used in the immunofluorescence assays.

| **Primary Antibody Against** | **Company** | **Cat. Number** | **Dilution** | **Secondary Antibody (dilution)** |
| --- | --- | --- | --- | --- |
| Oct4 | Invitrogen | A24867 | 4:20 | Alexa Fluor 555 anti-rabbit (1:250) |
| SSEA4 | Invitrogen | A24866 | 2:40 | Alexa Fluor 488 anti-mouse IgG3 (1:200) |
| TRA-1-60 | Invitrogen | A24868 | 2:40 | Alexa Fluor 555 anti-mouse IgM (1:200) |
| Sox2 | Invitrogen | A24759 | 2:40 | Alexa Fluor 488 anti-rat (1:200) |
| P75 | Advanced Targeting Systems | AB-N07 | 4:20 | Alexa Fluor 488 anti-mouse IgG (1:250) |
