# Supplementary figures and images for: Discovering the Potential of Dental Pulp Stem Cells for Corneal Endothelial Cell Production: A Proof of Concept
Source: Front Bioeng Biotechnol. 2021 Jan 28;9:617724. doi: 10.3389/fbioe.2021.617724 (PMC7876244; doi:10.3389/fbioe.2021.617724)

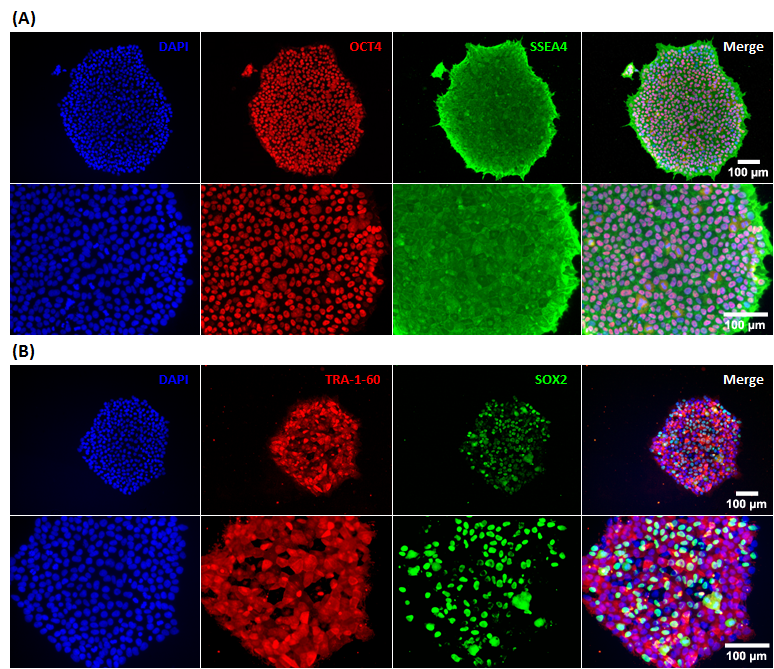

Supplement: Supplementary Figure 1 — Protein analysis of iPSC-derived from DPSC. Immunofluorescence stained images for reprogrammed cells from DPSC. Cells were positive for the pluripotent markers Oct4 (A-red), SSEA4 (A-green), TRA-1-60 (B-red), and Sox2 (B-green). Blue color corresponds to nucleus (DAPI). Scale bars: 100 μm. [file Image_1.TIF]

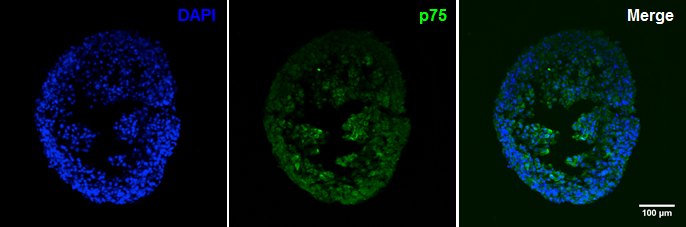

Supplement: Supplementary Figure 2 — Protein analysis of NCSC-derived from DPSC at day 4 of culture. Immunofluorescence stained images for dedifferentiated cells from DPSC. Neurospheres cells were positive for the NCSC markers p75. Blue color corresponds to nucleus (DAPI). Scale bar: 100 μm. [file Image_2.TIF]
